# Metagenomic characterization of sphingomyelinase C in the microbiome of humans and environments

**DOI:** 10.3389/fcimb.2022.1015706

**Published:** 2022-11-16

**Authors:** Jehyun Jeon, Seunghun Kang, Junho K. Hur, Mina Rho

**Affiliations:** ^1^ Department of Computer Science, Hanyang University, Seoul, South Korea; ^2^ Graduate School of Biomedical Science and Engineering, Hanyang University, Seoul, South Korea; ^3^ Department of Genetics, College of Medicine, Hanyang University, Seoul, South Korea; ^4^ Hanyang Institute of Bioscience and Biotechnology, Hanyang University, Seoul, South Korea; ^5^ Department of Biomedical Informatics, Hanyang University, Seoul, South Korea

**Keywords:** toxin, sphingomyelinase, metagenome, bacterial genome, active site

## Abstract

Bacterial sphingomyelinases (SMases) hydrolyze sphingomyelin and play an important role in membrane dynamics and the host immune system. While the number of sequenced genomes and metagenomes is increasing, a limited number of experimentally validated SMases have been reported, and the genomic diversity of SMases needs to be elucidated extensively. This study investigated the sequence and structural characteristics of SMases in bacterial genomes and metagenomes. Using previously identified SMases, such as the β-toxin of *Staphylococcus aureus*, we identified 276 putative SMases and 15 metagenomic SMases by a sequence homology search. Among the predicted metagenomic SMases, six non-redundant metagenomic SMases (M-SMase1−6) were selected for further analysis. The predicted SMases were confirmed to contain highly conserved residues in the central metal-binding site; however, the edge metal-binding site showed high diversity according to the taxon. In addition, protein structure modeling of metagenomic SMases confirmed structural conservation of the central metal-binding site and variance of the edge metal-binding site. From the activity assay on M-SMase2 and M-SMase5, we found that they displayed sphingomyelinase activity compared to *Bacillus cereus* SMase. This study elucidates a comprehensive genomic characterization of SMases and provides insight into the sequence-structure-activity relationship.

## Introduction

Bacterial toxins infect host cells (Edae, 2019). In particular, exotoxins such as hemolysin and leukocidin damage the host cell membrane by hydrolyzing plasma membrane lipids or forming pores (Vandenesch et al., 2012). Exotoxins produced by *Staphylococcus aureus* affect the host immune system and promote colonization and infection (Dinges et al., 2000; Vandenesch et al., 2012). β-hemolysins, also known as sphingomyelinases C (SMases), catalyze the hydrolysis of sphingomyelin (SM) to phosphocholine and ceramide (Goni and Alonso, 2002). Among the various types of SMases (Samet and Barenholz, 1999), bacterial SMases are structurally related to Mg^2+^-dependent neutral sphingomyelinase (nSMase), which has a wide range of optimum pH values (Rodrigues-Lima et al., 2000).

Bacterial SMases have been reported in several species, including *S. aureus*, *Bacillus cereus*, *Listeria ivanovii*, *Streptomyces griseocarneus*, *Helicobacter pylori*, *Leptospira interrogans*, and *Pseudomonas* sp. (Fujii et al., 1998; Fujii et al., 1999; Gonzalez-Zorn et al., 1999; Chan et al., 2000; Sueyoshi et al., 2002; Huseby et al., 2007; Sugimori, 2009; Narayanavari et al., 2012). Previous studies have characterized the function of bacterial SMases *via* mutagenesis of their enzymatic activities of hydrolysis, hemolysis, liposome disruption, and binding affinities (Tamura et al., 1995; Matsuo et al., 1996; Obama et al., 2003a; Obama et al., 2003b; Huseby et al., 2007; Oda et al., 2012b). Substitution of residues located at the metal-binding sites or close to the metal-binding site, from glutamic acid, aspartic acid, and histidine to alanine, asparagine, and glycine, respectively, resulted in a significant decrease in the hydrolyzing and hemolytic activities of SMase (Ago et al., 2006). In a structural study, the N16 and N197 residues in *B. cereus* SMase (Bc-SMase) formed a hydrogen bond with coordinating water molecules surrounding Co^2+^ ions at the metal-binding sites in the structure (Ago et al., 2006).

Binding sites for Mg^2+^, Mn^2+^, Co^2+^, and Ca^2+^ ions on SMases show highly conserved patterns (Ikezawa et al., 1986; Ago et al., 2006). For example, in the case of Bc-SMase, the central metal-binding sites include the carboxyl group of E53, imidazole nitrogen of H296, and coordinated waters bound to the two Co^2+^ ions, whereas the edge metal-binding sites include four residues, F55, N57, E99, and D100 (Ago et al., 2006). The four edge metal-binding residues phenylalanine, asparagine, glutamic acid, and aspartic acid are also well-conserved in other SMases, except for *L. ivanovii* SMase (Li-SMase), where asparagine is replaced with T92 (Openshaw et al., 2005). The residues at the central metal-binding site are also well conserved, except for G330 in *S. griseocarneus* SMase (Sg-SMase) (Fujisawa et al., 2021).

Bacterial SMases belong to the DNase I-fold superfamily and there are common feature in the active sites (Matsuo et al., 1996). However, a unique structural feature of bacterial SMases is the beta-hairpin, which includes aromatic amino acids and is distinct from DNase I enzymes (Openshaw et al., 2005; Ago et al., 2006; Huseby et al., 2007). Mutagenesis of two residues, W284 and F285, in the beta-hairpin apex of Bc-SMase resulted in significantly reduced binding affinity to SM liposomes and lower hemolysis of sheep erythrocytes (Ago et al., 2006). In addition, the aromatic beta-hairpin is involved in the cell membrane binding of SM liposomes together with a solvent-exposed loop (N93−P99 in Bc-SMase) (Ago et al., 2006).

SMases have been found in *L. ivanovii* isolated from ovine abortion and *B. cereus* isolated from bacteremia and endophthalmitis patients (Buchrieser et al., 2011; Oda et al., 2012a). An activity study on human cells revealed that *S. aureus* SMase (Sa-SMase) has a cytocidal effect on monocytes (Walev et al., 1996). Sa-SMase was also reported to promote cell damage and skin colonization of keratinocytes (Katayama et al., 2013). From a mode-of-action perspective, bacterial SMases allow pathogens to exert their virulence by interacting with the extracellular layer of the plasma membrane of eukaryotic cells. Changes in the membrane outer layer, induced by SMases, can disrupt the normal membrane-mediated signaling pathways that regulate several aspects of cellular functions, such as cell cycle, apoptosis, cytokine responses, and immune responses (Flores-Diaz et al., 2016), suggesting that the function of bacterial SMases is an important element in bacterial pathogenicity. Accordingly, several natural and synthetic SMase inhibitors have been studied to control bacterial infections (Oda et al., 2014). However, most genomic and activity studies on bacterial SMases have been performed on individual species. Considering the diversity of bacterial species, we anticipate that many unexplored bacterial SMases remain to be investigated, despite their potential clinical relevance.

This study investigated SMases in a comprehensive set of bacterial genomes and metagenomes and characterized the genomic patterns of SMases and expanded the SMase enzyme space with novel SMases in the Actinobacteria and Firmicutes groups. We also observed sphingomyelinase activity using fluorescence signal intensity and colorimetric assessment. Genomic and structural analysis with experimental validation enables us to expand our understanding of the sequence-structure-activity relationships of bacterial SMases.

## Materials and methods

### Preprocessing of the shotgun metagenome sequencing data

To identify SMases in the microbiome data, 31 previously sequenced samples were downloaded from the European Nucleotide Archive (ENA). They were collected from Jindo (two samples from two sites), Wando (four samples from four sites), Tongyeong (two samples from two sites), Taean (five samples from three sites), Asan (five samples from three sites), Korea (accession numbers: PRJEB48301 and PRJEB49194), and Shandon Sheng, China (13 samples from aquaculture systems, accession number: PRJEB22134). A total of 118 human gut and 119 human skin microbiome samples were obtained from the Human Microbiome Project Consortium (https://www.hmpdacc.org/, accession number: PRJNA48479) (Human Microbiome Project, 2012).

To filter out low-quality reads, the Sickle (v1.33) program (Joshi, 2011) was used with the options -q 20 -t sanger -l 60. Subsequently, the reads containing ambiguous “N” nucleotides were removed. The retained reads were assembled into contigs using MEGAHIT assembler (v1.0.3) (Li et al., 2016) with the default option, and the genes were predicted from the assembled contigs longer than 1,000 bp using FragGeneScan (v1.19) (Rho et al., 2010) with the options -w 1 -t complete.

### Identification of SMases in the microbiome

Previously identified SMases (Sa-SMase, Bc-SMase, Li-SMase, and Sg-SMase) were obtained through a literature survey (Openshaw et al., 2005; Ago et al., 2006; Huseby et al., 2007; Fujisawa et al., 2021). Accession numbers for the protein sequences of known SMases were P09978 (*S. aureus* RN4220), P11889 (*B. cereus* IAM 1029), Q9RLV9 (*L. ivanovii* ATCC 19119), and A6P7M9 (*S. griseocarneus*). To identify putative SMases, sequence homology searches were performed against known SMases using diamond-blastp (v0.9.14) (Buchfink et al., 2015) with the options –algo 0 –evalue 1e-10 –id 40 –query-cover 50 (70 for complete genomes) –subject-cover 50 (70 for complete genomes). Non-redundant SMases were obtained by clustering their sequences using the CD-HIT (Fu et al., 2012) program with the options -b 1000 -g 1 -G 0 -n 5 -c 0.98 -aL 1. Nucleotide and amino acid sequences of M-SMases are available in the supplementary material (**Supplementary Tables S1, 2**).

### Sequence alignments of SMases

To calculate the pairwise sequence identity of SMases, Clustal Omega (v1.2.4) (Sievers et al., 2011) was used with default options. Multiple sequence alignment was performed using MUSCLE (v.3.8.31) (Edgar, 2004) with the default option. The alignment result was visualized using ESPript (v.3.0) (Robert and Gouet, 2014) in expert mode.

### Embedding of SMases

To investigate the similarity of genomic patterns among SMases, *t*-distributed stochastic neighbor embedding (*t*-SNE) was performed using “Rtsne” in the R package (van der Maaten and Hinton, 2008) with a perplexity option of 20 and 1,000 iterations. Multiple sequence alignments of known and putative SMases were carried out using Clustal Omega (v1.2.4) (Sievers et al., 2011), and sequence identities against the known SMases were used as the feature values to learn their embedding.

### Protein structure modeling for metagenomic SMases

Protein structures of metagenomic SMases were predicted using the web-based structure modeling Phyre2 program (v.2.0) (Kelley et al., 2015) with the default option of normal modeling mode. The predicted model with the highest confidence score and percentage identity was selected. To visualize the protein structures, the PyMOL program (v.2.3.2) (Schrodinger, 2015) was used with the default options (cycles = 5 and cutoff = 2.0) of the *super* alignment method.

### Expression and purification of recombinant putative bacterial sphingomyelinase proteins

The deoxyribonucleic acid (DNA) fragments of the putative sphingomyelinase genes, i.e., M-SMase5 and M-SMase2, were synthesized (Integrated DNA Technologies, Coralville, IA, USA) and cloned into the pET28a expression plasmid. The plasmids containing the putative SMase genes were subsequently transformed into *Escherichia coli* BL21 (DE3) expression strain. The transformed cells were cultured in Luria Broth (LB) at 37°C until the culture reached an O.D_600_, and the recombinant protein was induced at 18°C for 48 h by the addition of isopropyl β-D-thiogalactoside (IPTG) (GenDEPOT, Baker, TX, USA) to a final concentration of 1 mM. Thereafter, the cell cultures were centrifuged at 20,000 × *g* for 30 min to remove the medium, and the pelleted cells were resuspended in lysis buffer (20 mM Tris-HCl (pH 8.0), 300 mM NaCl, 10 mM β-mercaptoethanol (BioRad, Hercules, CA, USA), 1% Triton X-100, and 1 mM phenylmethylsulfonyl fluoride (Sigma, St. Louis, MO, USA)) and lysed by sonication (Qsonica, Newtown, CT, USA). Cell debris was removed by centrifugation at 20,000 × *g* for 10 min, and the harvested soluble fraction was mixed with Ni-NTA resin (Takara, Nojihigashi, Japan) for purification. The mixture was incubated with stirring at 4°C in binding buffer (20 mM Tris-HCl (pH 8.0), 300 nM NaCl) for 1 h. After incubation, the Ni-NTA resins were washed with 10 volumes of the washing buffer. Next, the proteins bound to the Ni-NTA resin were eluted with elution buffer (20 mM Tris-HCl (pH 8.0), 300 nM NaCl, and 200 mM imidazole). Buffer exchange was then conducted on the eluted proteins with storage buffer (200 mM NaCl, 50 mM HEPES (pH 7.5), 1 mM DTT, and 40% glycerol (Sigma)) using an Amicon Ultra centrifugal filter unit (Millipore, Burlington, MA, USA). The purity of the recombinant proteins was confirmed by SDS-PAGE (10%) and Coomassie blue staining (BioRad), and the purified proteins were used for sphingomyelinase activity assays.

### Sphingomyelinase activity assay

The sphingomyelinase activities of the purified proteins were assessed using the Amplex Red Sphingomyelinase Assay Kit (Invitrogen, Carlsbad, CA, USA) by detecting the time-dependent fluorescence signal changes or colorimetric changes. The assays were conducted in a 200-μL reaction volume containing reagents provided in the kit and purified recombinant proteins (1 μM final concentration). The reaction mixtures were incubated at 37°C for 1 h for fluorescence studies. The fluorescence signal (λ_exc_ = 584 nm; λ_em_ = 612 nm) was measured at 1 min intervals for 1 h by Fluoroskan™ Microplate Fluorometer (Thermo-Fisher, Massachusetts, USA). For a positive control, we utilized the recombinant *B. cereus* sphingomyelinase provided in the kit. For a negative control, we prepared recombinant His-tagged *Streptococcus pyogenes* Cas9 as an irrelevant protein. The sphingomyelinase activities were also assessed by comparing the colorimetric changes of the reactions after the samples were incubated at 37°C for 1 h.

### Hemolytic activity assay using blood agar plate

The hemolysis activities of sphingomyelinase were evaluated by using blood agar plates containing sheep blood. First, *E. coli* BL21 (DE3) was transformed with M-SMase5 and M-SMase2 expression vectors, and cultured at 37°C until the culture reached an OD_600_ of 0.6. The cells were subsequently induced to express the recombinant proteins by adding IPTG to the final concentration of 1 mM. The cells were induced at 18°C for 18 h. The induced *E. coli* cultures were inoculated on blood agar plates (Kisan-biotech, Seoul, Korea) and the plates were incubated at 37°C for 18 h. The changes of the red color of the blood agar plates indicated the hemolysis activities of the expressed recombinant proteins.

## Results

### Sphingomyelinases C in bacterial genomes and metagenomes

To explore SMases in bacterial genomes, 16,613 complete genomes were obtained from the NCBI repository (downloaded in Mar 2020). Their protein sequences were queried against the protein sequences of previously identified SMases (Openshaw et al., 2005; Ago et al., 2006; Huseby et al., 2007; Fujisawa et al., 2021). A total of 276 putative SMases were identified (**Table 1**). At the phylum level, Firmicutes (Bacillota), Actinobacteria (Actinomycetota), and Cyanobacteria accounted for 61.9, 37.7, and 0.4%, respectively. *Bacillus* (31.5%), *Staphylococcus* (29%), and *Listeria* (1.4%) were the major genera in Firmicutes, whereas *Streptomyces* (32.6%) was the dominant genus in Actinobacteria.

**Table 1:** Putative SMases identified from bacterial complete genomes.

Phylum	Genus	Number of SMases	SMases (%)	Seq. id. with known SMases (%)			
				Sg-SMase	Bc-SMase	Sa-SMase	Li-SMase
Actinobacteria	*Streptomyces*	90	32.61	75.5^*^	46.7	46.2	44.6
	*Nocardia*	3	1.09	73.6	48.4	46.3	44
	*Kitasatospora*	3	1.09	68.9	45.5	45.3	42.4
	*Salinispora*	2	0.72	55.5	50.5	45.1	45.6
	*Saccharothrix*	1	0.36	48.2	46.9	48.2	46.6
	*Arthrobacter*	1	0.36	43.2	44.9	n/a	40.6
	*Luteipulveratus*	1	0.36	52.2	46.1	44.5	45.2
	*Lentzea*	1	0.36	52.3	49.1	45.7	46.6
	*Nocardiopsis*	1	0.36	54.2	42.7	44.1	43.9
	*Allokutzneria*	1	0.36	44.6	n/a	41.4	42.7
Cyanobacteria	*Nostoc*	1	0.36	47.8	59.4	56.7	54.1
Firmicutes	*Bacillus*	87	31.52	46.4	94.9	58.5	55.7
	*Staphylococcus*	80	28.99	45.6	58	87.5	56.2
	*Listeria*	4	1.45	45.5	55	55.8	98.2

^*^The sequence identity was obtained by local sequence alignment using diamond-blastp (v0.9.14).

n/a indicates that we could not find any hit in diamond-blastp search.

From 268 human and environmental microbiomes, 15 metagenomic SMases were identified: three from aquaculture farms, one from aquaculture systems, and 11 from human skin (**Supplementary Table 3**). Among the 11 skin metagenomic SMases, one gene was identical to the putative SMase of *S. aureus*, and 10 genes were homologous with putative SMases of *S. epidermidis*, showing an average sequence identity of 98.86%. After clustering these 15 metagenomic SMases with the threshold of 98% protein sequence similarity, six clusters were constructed. We randomly selected a SMase from each cluster to obtain six non-redundant SMases (M-SMase1–6) for further studies (**Table 2**).

**Table 2:** Putative SMases identified from human skin and marine aquaculture microbiomes.

SMase	Isolation source	Length (aa)	Homologous protein (accession number)	Source organism of homologous protein^1^	Seq. id. (%)	Seq. id. with known SMases (%)			
						Sg-SMase	Bc-SMase	Sa-SMase	Li-SMase
M-SMase1	aquaculture system	525	sphingomyelin phosphodiesterase (CCK76853.1)	*O. antarctica* RB-8	78.1^2^	40.8	49.7	50	47.3
M-SMase2	aquaculture farm	524	sphingomyelin phosphodiesterase (CCK76853.1)	*O. antarctica* RB-8	75.5	42.8	49.3	47.2	48
M-SMase3	aquaculture farm	527	sphingomyelin phosphodiesterase (CCK76853.1)	*O. antarctica* RB-8	85.4	41.2	46.4	46.3	45.8
M-SMase4	aquaculture farm	526	sphingomyelin phosphodiesterase (CCK76853.1)	*O. antarctica* RB-8	78.2	42.2	47.8	48.8	47.5
M-SMase5	human skin (anterior nares)	330	sphingomyelin phosphodiesterase (WP_001652363.1)	*S. aureus*	100	45.8	58.9	95.7	56.4
M-SMase6	human skin (retroauricular crease)	334	sphingomyelin phosphodiesterase (MBM0781907.1)	*S. epidermidis*	100	n/a	52	52.4	54.2

^1^Homologous protein was identified from the web-based BLAST program.

^2^The protein sequence identity was obtained by local sequence alignment using diamond-blastp (v0.9.14).

**Figure 1:**
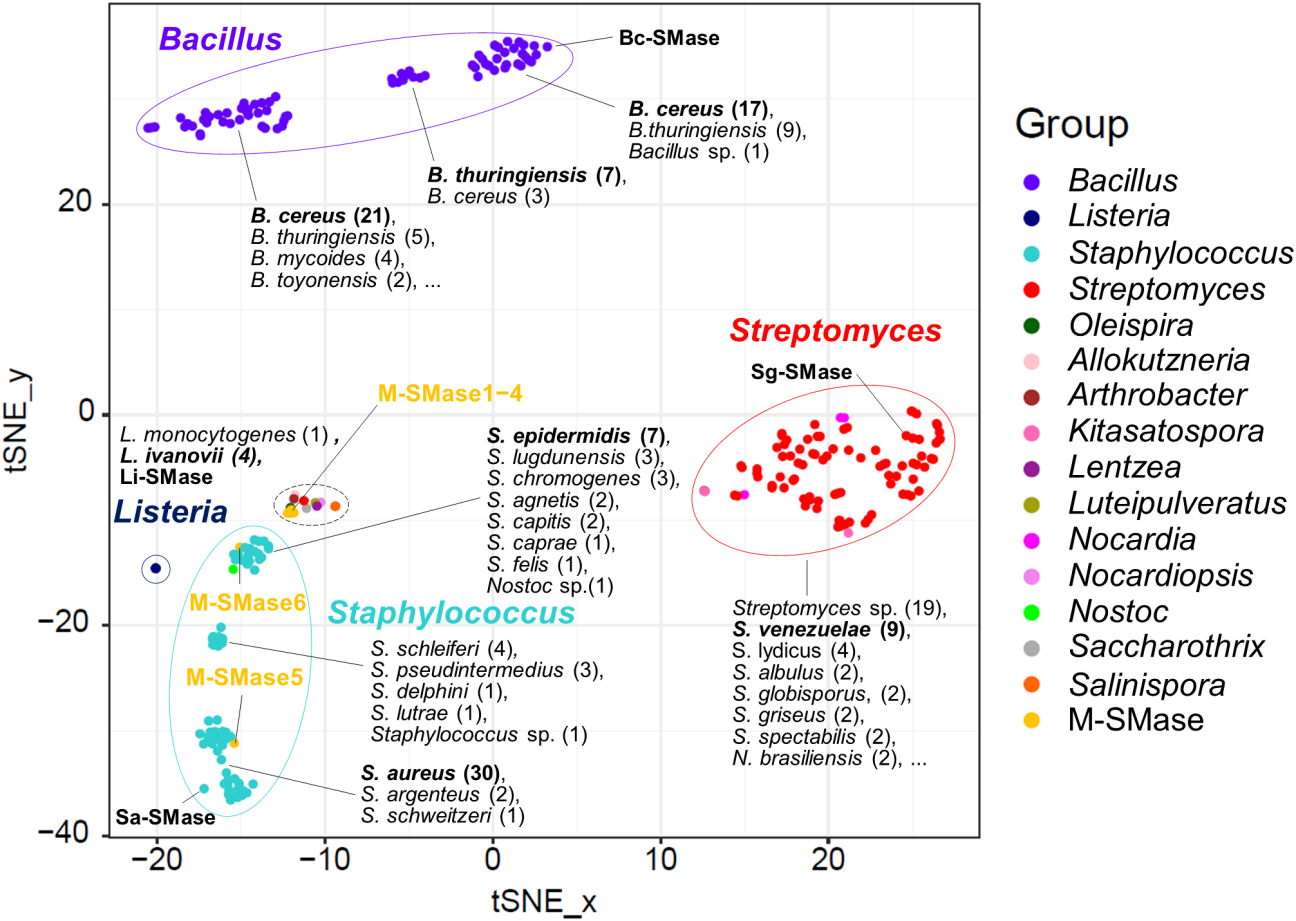
Embedding for genomic diversity of bacterial and metagenomic SMases. Protein sequence identity with four known SMases was used as a feature of *t*-SNE. M-SMase; metagenomic sphingomyelinase C. The number of SMases originated from a species is provided in the parentheses. A black dashed circle indicates the following species: *Allokutzneria albata* (1), *Lentzea guizhouensis* (1), *Luteipulveratus mongoliensis* (1), *Nocardiopsis gilva* (1), *Oleispira antarctica* (1), *Psychromicrobium lacuslunae* (1), *Saccharothrix espanaensis* (1), *Salinispora arenicola* (1), *Salinispora tropica* (1), and *Streptomyces ficellus* (1).

When known and putative SMases were represented in the embedding space using *t*-SNE, SMases were clustered by taxonomic group (**Figure 1**). This result implies that the genomic patterns of the SMases are reflected in the embedding space. The median pairwise sequence identities of SMases in the same genus were 92, 96, 68, and 72% for *Bacillus*, *Listeria*, *Staphylococcus*, and *Streptomyces*, respectively (**Supplementary Figure 1**). In particular, SMases belonging to the same species showed a high sequence identity (>90%; **Supplementary Figure 1**). Notably, putative SMases originated from *B. cereus* and *B. thuringiensis* were divided into three groups, and Bc-SMase was clustered with 17 putative SMases of *B. cereus* in the embedding space (**Figure 1**).

While M-SMase1−4, identified from marine aquaculture farms, showed weak homology with four known SMases (sequence identity ranging from 37% to 44%; **Table 2**), they are similar to each other (ranges from 76% to 84%). However, M-SMase1−4 were closely located to the putative SMase identified from the complete genome of *Oleispira antarctica* RB-8 in the embedding space and showed an average percent identity of 79.29% (**Figure 1** and **Supplementary Table 3**). M-SMase5−6 identified from human skin showed 95.7% and 52.4% protein sequence identities with Sa-SMase, respectively, and were tightly clustered in the embedding space (**Figure 1** and **Table 2**). However, we found that M-SMase5 and 6 were identical to the putative SMase of *S. aureus* and *S. epidermidis*, respectively, that we identified from the complete genomic sequences.

### Characteristics of the sphingomyelinase C sequences and structures

The metal-binding sites in known SMases are distinctively represented in their amino acid sequences. In 276 putative SMases identified from the bacterial genomes, all the central metal-binding residues showed a conservation ratio of over 99% compared to known SMases (**Supplementary Table 4**). In addition, the edge metal-binding residues also showed a conservation ratio of over 99%, except for *Staphylococcus* species. Putative SMases of *Staphylococcus* were predicted from various species, and the sequence pattern for the edge metal-binding residues differed according to species; however, the pattern in each species was highly conserved (**Supplementary Table 5**). The phenylalanine residue in the edge metal-binding site (F55 in Sa-SMase) was 100% conserved in all *Staphylococcus* species; however, asparagine, glutamic acid, and aspartic acid residues (N57, E98, and D99 in Sa-SMase) were only present in *S. argenteus*, *S. aureus*, *S. lutrae*, *S. schleiferi*, and *S. schweitzeri* (**Supplementary Table 5**). In addition, these residues were replaced with lysine, valine, and serine in *S. capitis*, *S. caprae*, and *S. epidermidis*, and proline, glutamic acid, and asparagine in *S. delphini* and *S. pseudintermedius*, respectively (**Supplementary Table 5**).

**Figure 2:**
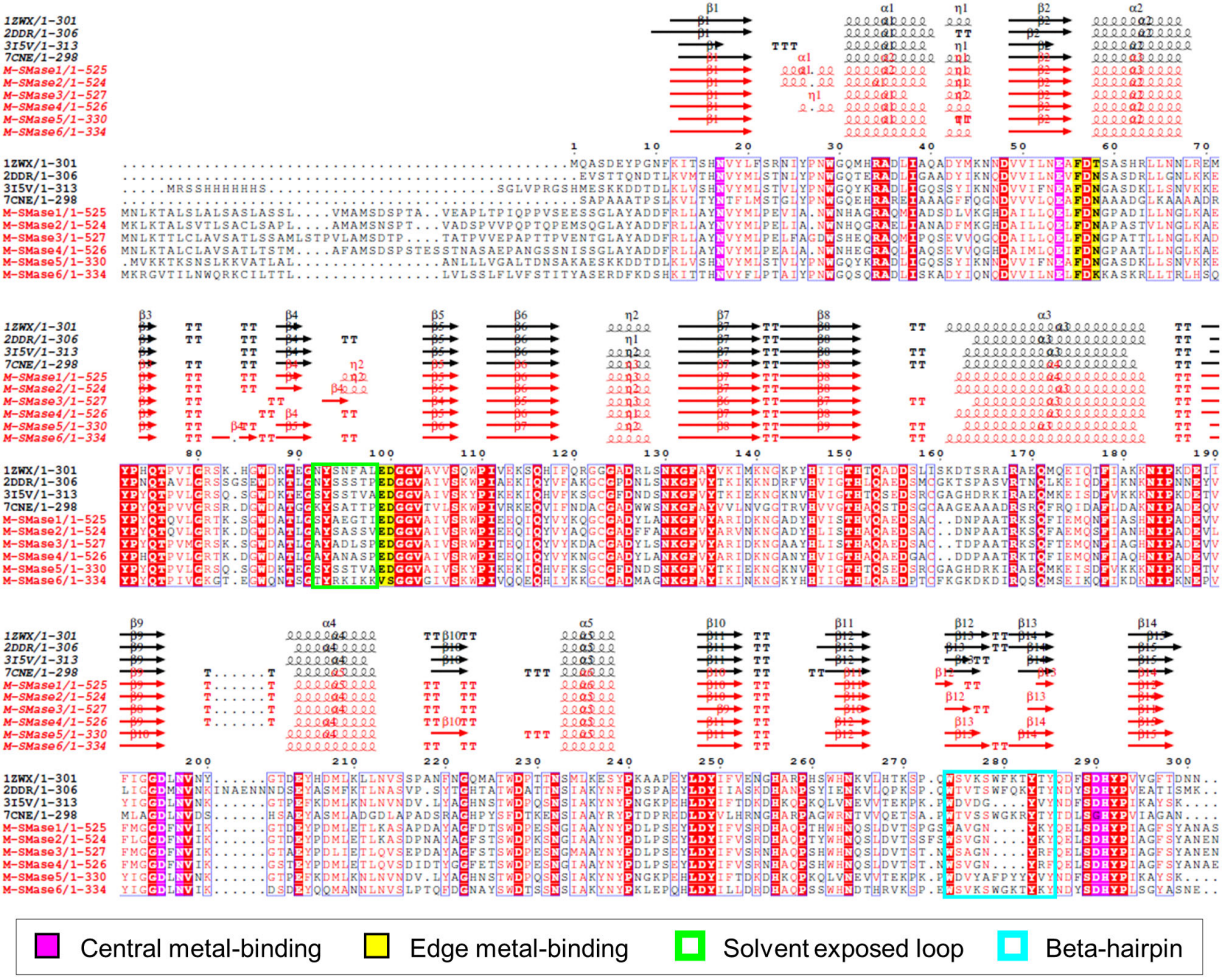
Multiple sequence alignment of known and metagenomic SMases (M-SMase1−6). Known SMases are represented with the following PDB codes: 1ZWX for Li-SMase, 2DDR for Bc-SMase, 3I5V for Sa-SMase, and 7CNE for Sg-SMase. Protein structures of known SMases obtained from the PDB database are depicted in **Supplementary Figure S3**. Identical residues are boxed in red. Metagenomic SMases are labeled with a red color. Note that 3I5V is not a naturally occurring protein sequence but is a His-tagged version at the N-terminus.

To investigate the sequence characteristics of bacterial and metagenomic SMases, the protein sequences of four known SMases (Li-SMase, Bc-SMase, Sa-SMase, and Sg-SMase) and M-SMase1−6 were compared (**Figure 2**). Multiple sequence alignment revealed that the essential residues for enzymatic activity were highly conserved in these sequences (**Figure 2**). In particular, six residues involved in central metal-binding were identical in all the M-SMases. Note that Sg-SMase is an exception to G330 (PDB code 7CNE). Because the glycine residue (G330 in 7CNE) could not form a hydrogen bond with a metal-coordinating water molecule in Sg-SMase, the arginine residue (R278 in 7CNE) formed a hydrogen bond with a coordinating water molecule (Fujisawa et al., 2021).

In edge metal-binding residues, the residues in the active site were well-conserved, except for M-SMase6 (**Figure 2**). The edge metal-binding site of M-SMase6 was substituted with valine and serine residues, instead of glutamic acid and aspartic acid (E99 and D100 in Bc-SMase). This pattern was also observed in putative SMases of *S. capitis, S. caprae*, and *S. epidermidis* identified from the bacterial genomes. Notably, the Li-SMase (92T in 1ZWX) varied among the known SMases.

While major alpha-helices and beta-strands were highly conserved in M-SMases, M-SMase1−2 and M-SMase4 had an additional alpha-helix in the N-terminal region of the modeled structures (between β1 and α1 in Li-SMase) (**Figure 2**). In the C-terminal region, M-SMase1−4 had ~190 aa more residues than those of the known SMases (**Supplementary Figure 2**). In addition, the putative SMase of *O. antarctica*, which is the protein most similar to M-SMase1−4, also had additional sequences in the C-terminal region. The additional sequences of M-SMase1−4 showed an average of 87% sequence identity to each other. However, they did not match any other functional proteins except for SM phosphodiesterase from a homology search against the non-redundant protein database. In addition, functional domain searches using Pfam and Conserved Domain Database (CDD) did not show any hits, and their biological functions are yet to be revealed.

### Structural comparison of bacterial and metagenomic sphingomyelinases C

**Figure 3:**
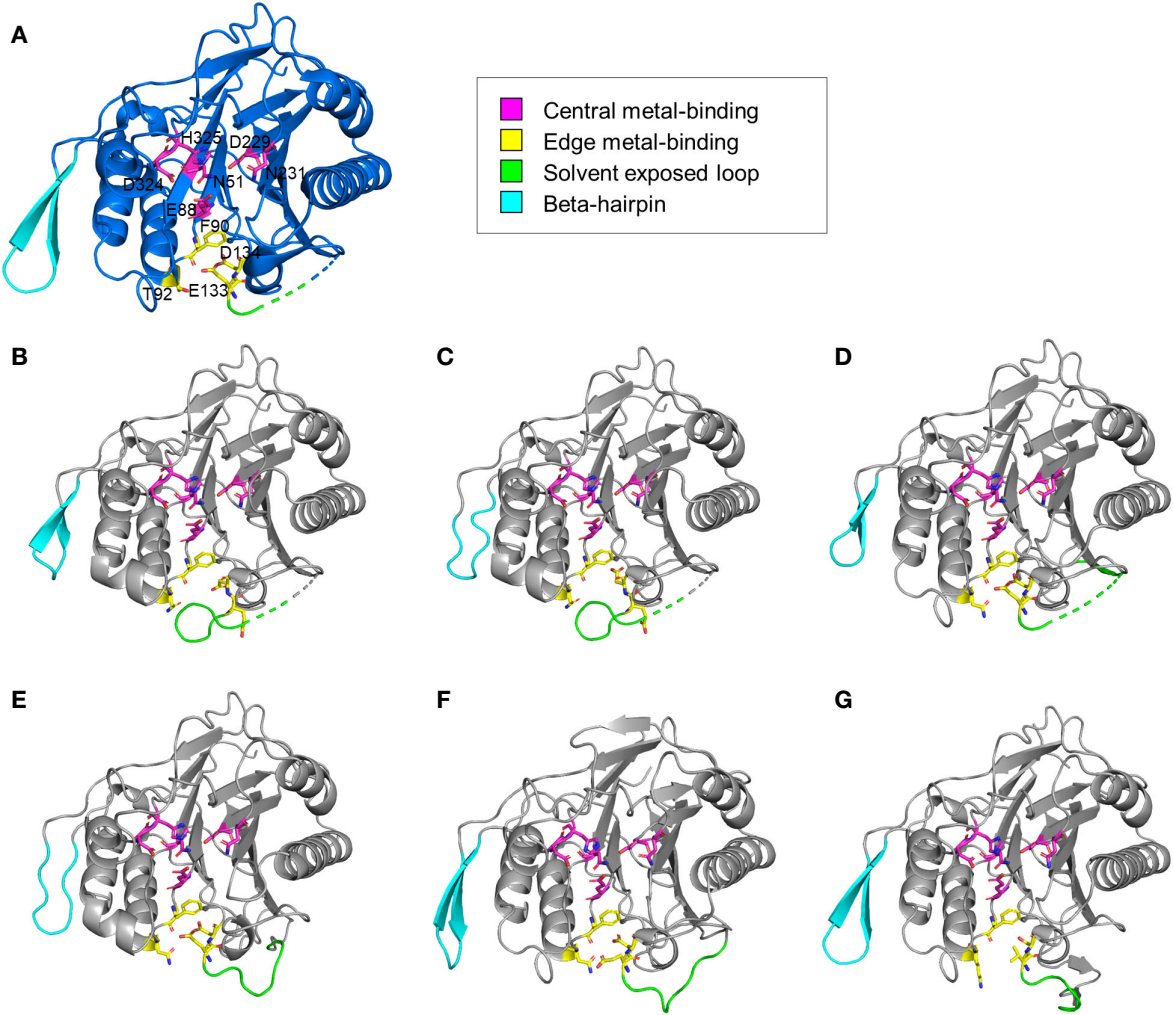
Structural comparison of known and metagenomic SMases (M-SMase1−6). **(A)** Protein structure of Li-SMase (PDB code 1ZWX) used as the template in superposition. **(B–G)** Modeled protein structures of M-SMase1−6. **(B)** M-SMase1, **(C)** M-SMase2, **(D)** M-SMase3, **(E)**, M-SMase4, **(F)** M-SMase5, **(G)** M-SMase6.

The structural features of the newly identified M-SMases were investigated using the protein structures of known SMases. Structurally modeled protein structures of M-SMase1−6 were compared with those of previously identified SMases to analyze their structural characteristics (**Figure 3**). Compared with known SMases, the structural homology analysis of M-SMase1−3 and M-SMase6 showed root mean square deviation (RMSD) values of 1.074, 1.231, 0.731, and 2.005, respectively, for Li-SMase. M-SMase4−5 showed RMSD values of 1.911 and 0.471, respectively, for Sa-SMase.

Consistent with known SMases, residues involved in metal-binding were highly conserved in the modeled structures of M-SMase1−6. However, in contrast to other SMases, the glutamic acid residue in the edge metal-binding site (E99 in Bc-SMase) of M-SMase1−2 was flipped (**Figures 3B−C**). Mutagenesis of the edge metal-binding residues N57, E99, and D100 in Bc-SMase resulted in decreased binding affinity to sheep erythrocytes (Oda et al., 2012b). Although flipping of the glutamic acid residue at the edge metal-binding site has not yet been revealed, this result suggests that they might represent weak binding to membranes compared with the known SMases.

A beta-hairpin structure was found in only four M-SMases (M-SMase1, M-SMase3, M-SMase5, and M-SMase6) (**Figure 3**). The length of the beta-hairpin in M-SMase1 and M-SMase3 was four aa shorter than the template (Li-SMase), and that of M-SMase5−6 was identical to that of the template. In addition, while beta-hairpin apex residues between the two beta-strands of known SMases (β12−β13 in Li-SMase) were hydrophobic amino acids tryptophan and phenylalanine (W313 and F314 in Li-SMase), those of M-SMase1−6 were substituted with uncharged residues such as glycine and asparagine. In a previous mutagenesis study, binding affinity to SM liposomes was reduced, and disruption of SM liposomes and sheep erythrocytes was decreased when beta-hairpin apex residues tryptophan and phenylalanine were substituted with alanine (Ago et al., 2006). This result suggests that while M-SMases could catalyze the hydrolysis of SM, they might have a low binding affinity to SM liposomes and lower hemolytic activity compared with the known SMases.

### Experimental activity assessments of putative bacterial sphingomyelinases C

To assess the sphingomyelinase activity and hemolytic activity of the predicted M-SMases, genes were synthesized from the sequence information of the two putative M-SMase DNA sequences (M-SMase5 and M-SMase2), and the recombinant proteins were prepared. The His-tagged M-SMases from *E. coli* (**Supplementary Figure 4**) were expressed, purified, and analyzed by sodium dodecyl-sulfate polyacrylamide gel electrophoresis (SDS-PAGE), which confirmed the anticipated molecular weights of 36.7 kDa and 61.3 kDa of M-SMase5 and M-SMase2 proteins, respectively.

**Figure 4:**
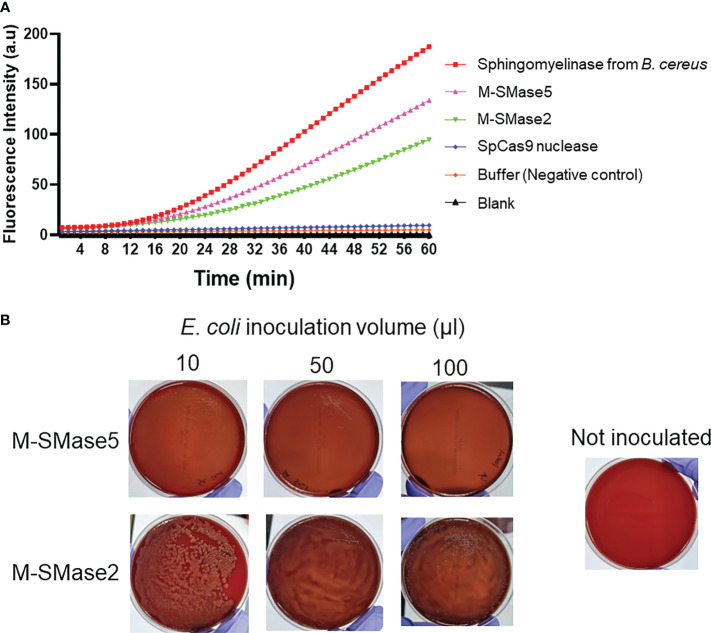
Biochemical and functional assessment of sphingomyelinase activities of predicted bacterial M-SMases. **(A)** 60-minute time course of fluorescence signal intensity changes by the recombinant proteins in sphingomyelinase activity assays. The time-dependent changes in the fluorescence intensities indicate sphingomyelinase activities. Recombinant M-SMase5 and M-SMase2 are putative sphingomyelinase proteins. Sphingomyelinase from *Bacillus cereus* (*B. cereus*) and *Streptococcus pyogenes* Cas9 (SpCas9) nuclease proteins are included as positive and negative controls, respectively. **(B)** Blood agar plate hemolysis assays to assess the hemolytic function of the putative sphingomyelinase proteins. *E. coli* cells that express recombinant M-SMase5 and M-SMase2 proteins were inoculated onto blood agar plate. The clearing of the red color indicates the extent of hemolysis.

To assess the sphingomyelinase activities of the recombinant proteins, fluorescence-based assays were carried out (**Figure 4A**). In the assay, fluorescence-quenched SM substrates were incubated with the purified proteins, and the time-dependent changes in the fluorescence signals were quantified. We anticipated that the protein-dependent cleavage of the substrates would increase the fluorescence signals. As a positive control, purified *B. cereus* sphingomyelinase protein was used (Nishiwaki et al., 2004). As a negative control, *S. pyogenes* Cas9 nuclease protein without sphingomyelinase activity was used. The assay showed that both M-SMase2 and M-SMase5 induced significant enhancements in the fluorescence signal, comparable to that of *B. cereus* sphingomyelinase. In contrast, changes in fluorescence signal were not detected for *S. pyogenes* Cas9 nuclease or buffer without recombinant proteins. We also conducted colorimetric assessments of M-SMase2 and M-SMase5 for sphingomyelinase activity (**Supplementary Figure 5**). Significant color changes were observed for M-SMase2, M-SMase5, and sphingomyelinase from *B. cereus* provided by the assay kit. The changes in the color were comparable to that observed for hydrogen peroxide used as a positive control. Both the fluorescence and colorimetric results consistently suggested that the recombinant M-SMase2 and M-SMase5 have sphingomyelinase activities.

The hemolysis activity of M-SMase5 and M-SMase2 proteins was also assessed on blood agar plates (**Figure 4B**). For this purpose, we transformed BL21(DE3) *E. coli* protein expression strain with vectors containing M-SMase5 and M-SMase2, and inoculated the cells on the blood agar plate. If the recombinant protein increases the hemolysis activity, the blood agar plate becomes more transparent with concomitant loss of the red color. The results in **Figure 4B** confirmed a significant increase in transparency and a notable reduction of red color with cells expressing M-SMase5. In contrast, the plate inoculated with cells expressing M-SMase2 showed only a moderate increase in transparency. The seemingly higher hemolysis activity of M-SMase5 is consistent with the higher expression level of the recombinant M-SMase5 in *E. coli* compared to that of M-SMase2 (**Supplementary Figure 4**).

## Discussion

Bacterial toxins have drawn attention because of their risks to public health and many guidelines and regulations have been applied to prevent the pathogens that produce them from spreading in the community (Forbes, 2020). SMase, a bacterial toxin, has been studied for its virulence and potential pathogenicity to determine its enzymatic function in damaging the host cell membrane. However, these studies have focused on a specific species (Fujii et al., 1998; Fujii et al., 1999; Gonzalez-Zorn et al., 1999; Chan et al., 2000; Sueyoshi et al., 2002; Huseby et al., 2007; Sugimori, 2009; Narayanavari et al., 2012) and did not have the opportunity to find a more diverse bacterial origin to expand the SMase enzyme space. This study investigated SMase enzymes in genomes and metagenomes obtained from various environments, including humans. We identified 276 and 15 putative SMases from bacterial isolate genomes and environmental microbiomes, respectively, characterized their sequence and structural features with respect to enzymatic activity, and experimentally confirmed the SMase activity of two M-SMases.

Compared with known SMases, the newly identified SMases exhibited highly conserved patterns of active sites and metal-binding sites. Although SMase showed a taxonomic bias of the host bacteria, including Actinobacteria, Cyanobacteria, and Firmicutes, various genera were found in Actinobacteria in addition to the known genus *Streptomyces*. While most central metal-binding residues showed a high conservation ratio of over 99%, species-specific sequence patterns were observed for edge metal-binding residues, particularly in *Staphylococcus* species. These results suggest that the cofactor and substrate scope of SMases can be expanded by new SMases.

Few studies are currently available on the sphingomyelinase activities of the metagenomic SMases. As an experimental validation of our prediction, we prepared recombinant proteins based on the sequences of M-SMase2 and M-SMase5, and conducted biochemical assessments of their sphingomyelinase activities. We utilized a fluorescence-based sphingomyelinase assay from a previous study that investigated the activity of recombinant *B. cereus* sphingomyelinase protein (Nishiwaki et al., 2004). The time-dependent changes in the fluorescence signal by M-SMases2 and M-SMase5 were similar to that of *B. cereus* sphingomyelinase. In addition, significant color changes were also observed for these samples.

The hemolytic capacity of M-SMase2 and M-SMase5 were also assessed by inoculating blood agar plates with *E. coli* cells transformed with vectors to express the recombinant proteins. We observed significantly enhanced transparency in the M-SMase5 plate, and moderately increased transparency in M-SMase2 plate. The difference in the extent of plate clearing was consistent with the results from the fluorescence-based assay: M-SMase5 showed higher sphingomyelinase activity than M-SMase2. Collectively, our findings suggest that the metagenomic M-SMase2 and M-SMase5 are *bona fide* sphingomyelinases, and their expression in bacterial cells increases hemolytic activities.

This study established a foundation for screening bacterial SMases in the environment by comprehensively performing genomic and structural analyses and an activity assay. One of the notable findings in the metagenomic analyses was that the *O. antarctica*-like SMase contains additional C-terminal regions of unknown biological function, which may be related to their functions being distinct from other known SMases. In addition, some key residues in the beta-hairpin apex have the potential to regulate the affinity of SMases to SM. In summary, the results of this study suggest that further characterization of the novel SMases and experimental confirmation, such as substrate-binding affinity and hemolytic activity, will provide more information regarding the diverse functions of the SMase enzyme group.

## Data availability statement

The original contributions presented in the study are included in the article/**Supplementary Material**. Further inquiries can be directed to the corresponding authors.

## Author contributions

MR and JH designed the study. JJ and MR performed bioinformatic analysis. SK and JH performed experimental activity assessment. All authors wrote, read, and approved the final manuscript.

## Funding

This work was supported by the Collaborative Genome Program of the Korea Institute of Marine Science and Technology Promotion (KIMST) funded by the Ministry of Oceans and Fisheries (MOF) [No. 20180430], a grant from National Research Foundation of Korea (NRF) [No. 2020R1I1A2075393 and 2021M3A914024452] and the BK21 FOUR (Fostering Outstanding Universities for Research) project of the National Research Foundation of Korea Grant.

## Conflict of interest

The authors declare that the research was conducted in the absence of any commercial or financial relationships that could be construed as a potential conflict of interest.

## Publisher’s note

All claims expressed in this article are solely those of the authors and do not necessarily represent those of their affiliated organizations, or those of the publisher, the editors and the reviewers. Any product that may be evaluated in this article, or claim that may be made by its manufacturer, is not guaranteed or endorsed by the publisher.
