# Supplementary material for: Metagenomic characterization of sphingomyelinase C in the microbiome of humans and environments
Source: Front Cell Infect Microbiol. 2022 Nov 16;12:1015706. doi: 10.3389/fcimb.2022.1015706 (PMC9710629; doi:10.3389/fcimb.2022.1015706)
Supplement: Supplementary file 1 [file DataSheet_1.docx]

Supplementary Material

# Supplementary Figures and Tables

## Supplementary Figures


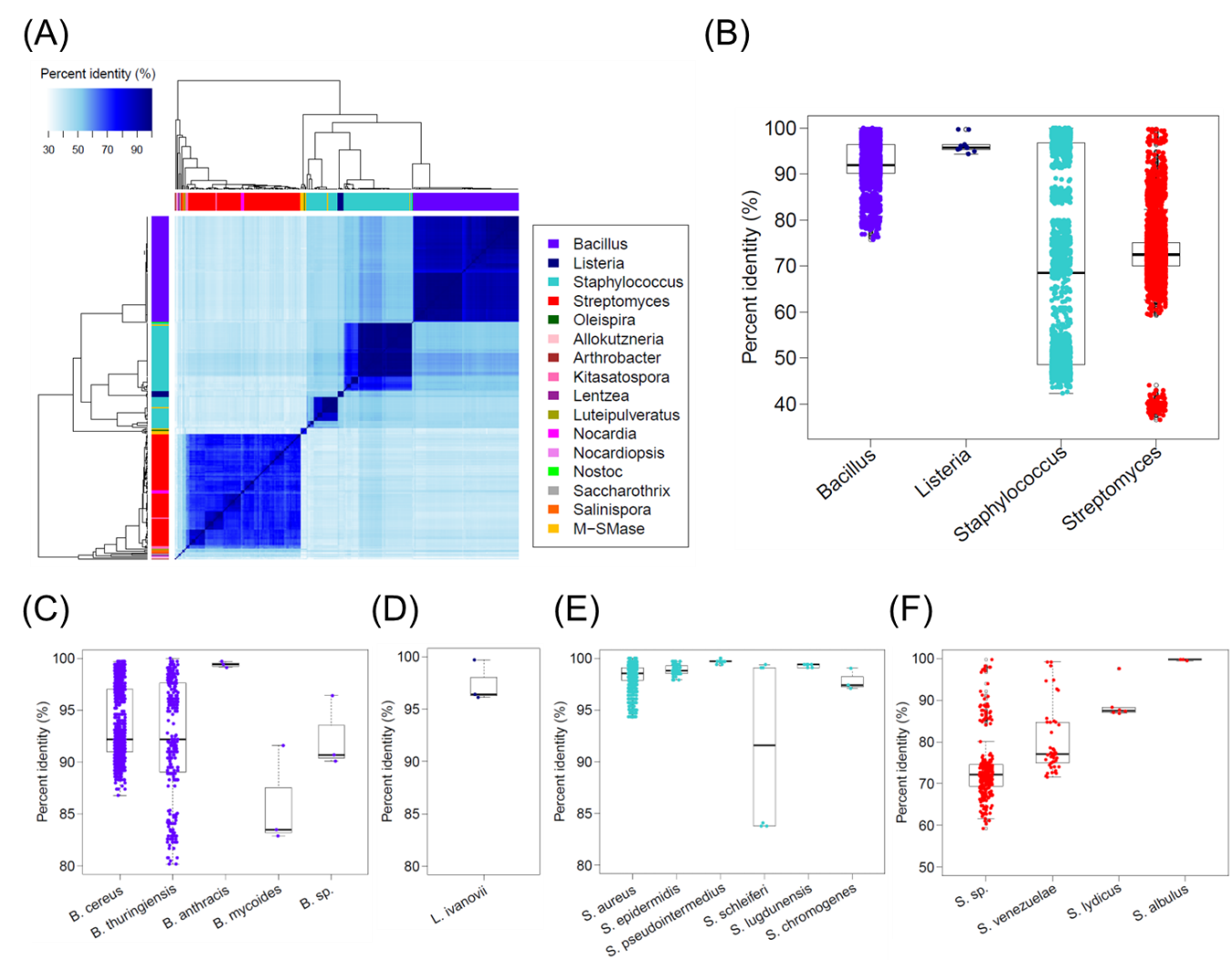


**Supplementary Figure 1.** Pairwise sequence identities of SMases between intra- and inter-genus. (A) Inter-genus sequence identities of known and putative SMases. (B) Intra-genus sequence identities of four genera SMases. (C)−(F) Intra-species sequence identities of four genera SMases. The number of species more than 3 were visualized in the boxplot. (C) *Bacillus*, (D) *Listeria*, (E) *Staphylococcus*, (F) *Streptomyces* species.


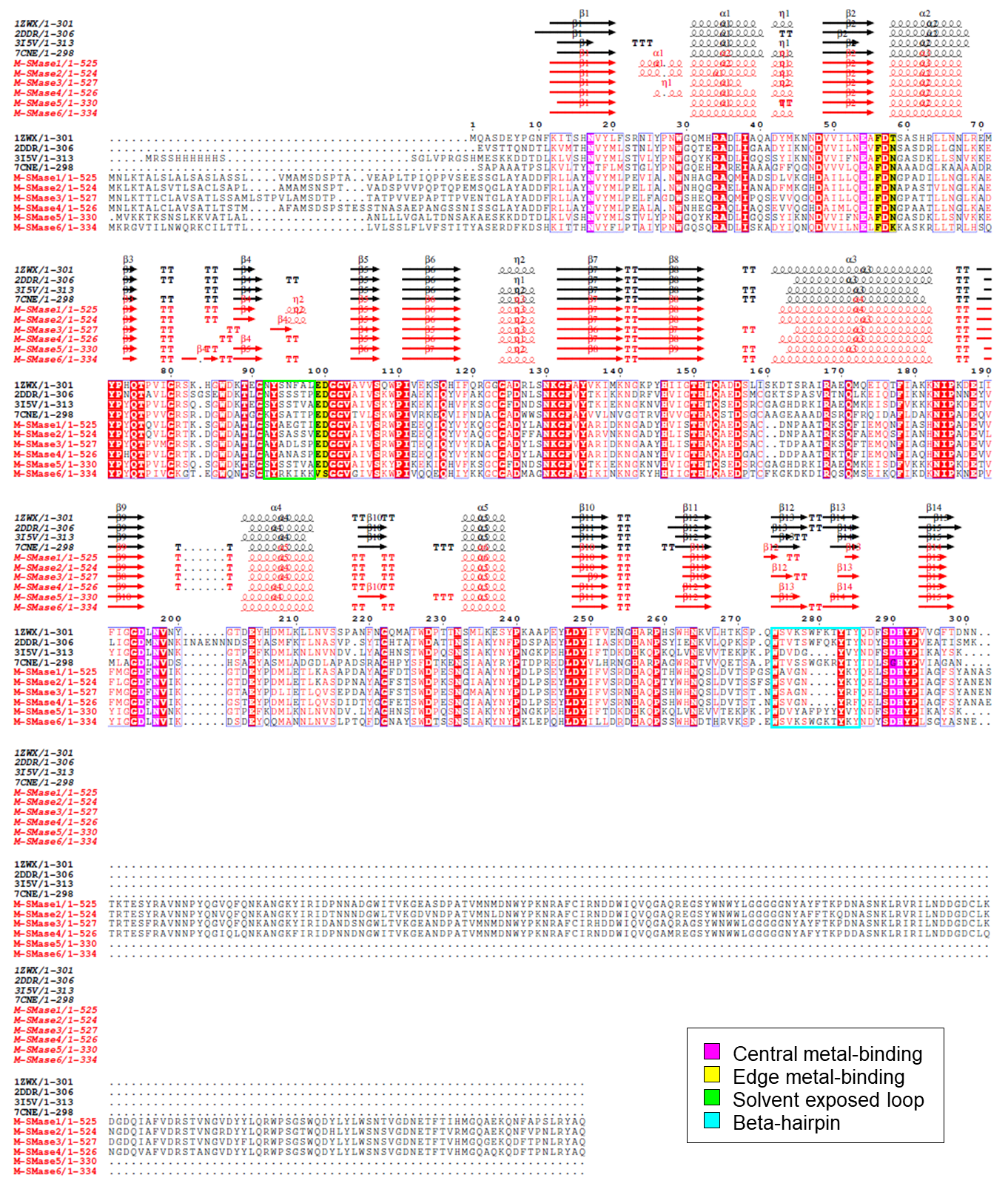


**Supplementary Figure 2.** Multiple sequence alignment of known and metagenomic SMases (M-SMase1−6). Identical residues are boxed in red. Metagenomic SMases are labeled with the red color.


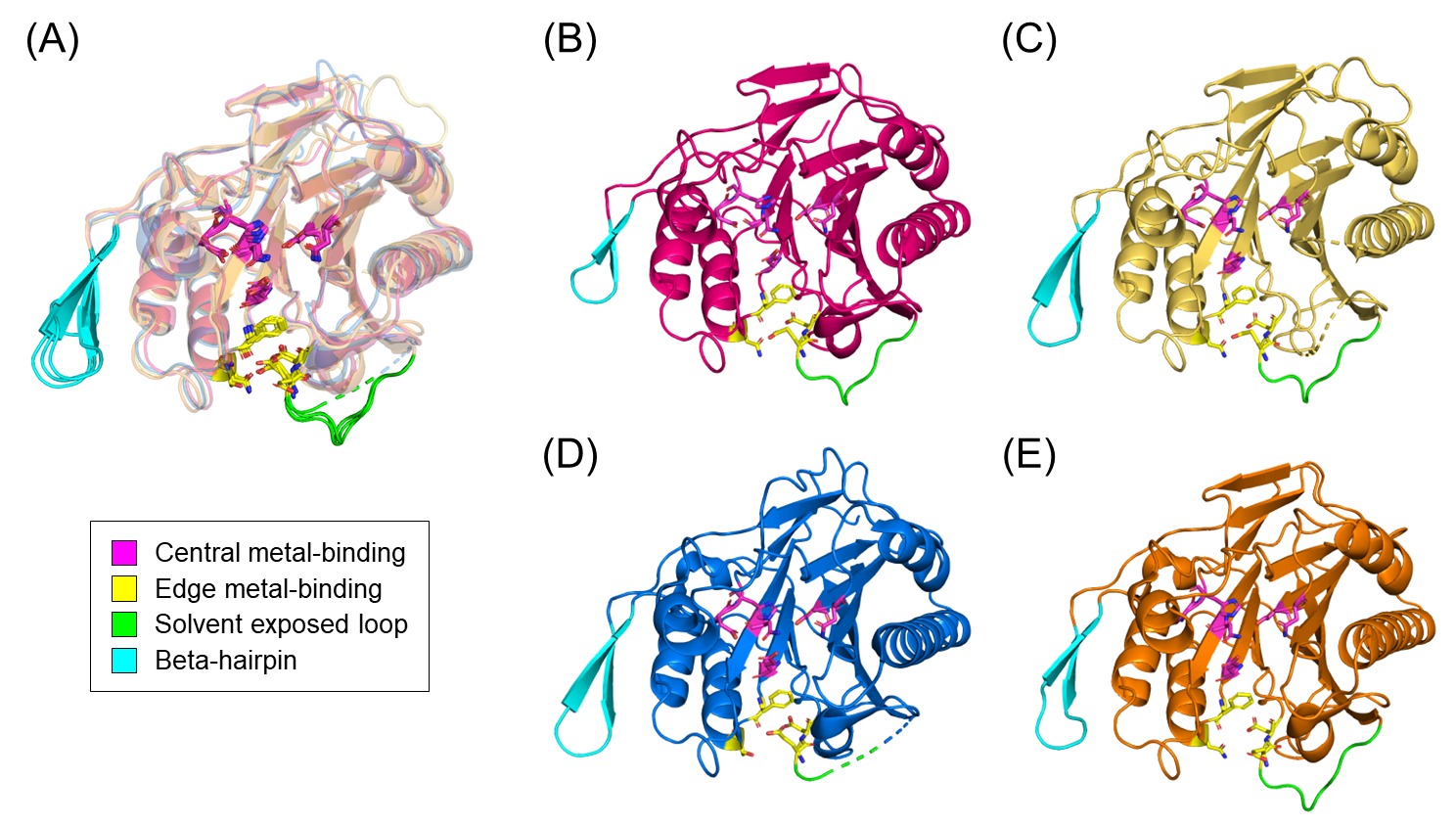


**Supplementary Figure 3.** Three-dimensional protein structures of known SMases. (A) Superimposed protein structures of known SMases. (B) Sa-SMase (PCB code: 3I5V), (C) Bc-SMase (PDB code: 2DDR), (D) Li-SMase (PDB code: 1ZWX), (E) Sg-SMase (PDB code: 7CNE).

**Supplementary Figure 4.** Protein size assessment of recombinant putative sphingomyelinase proteins. The sizes of the recombinant His-tagged proteins based on the predicted M-SMase-5 and M-SMase-2 were assayed by sodium dodecyl sulphate – polyacrylamide gel electrophoresis (SDS-PAGE) and Coomassie stain. The anticipated protein sizes are indicated as red arrows.

**Supplementary Figure 5.** Colorimetric assessment of biochemical activities of predicted bacterial sphingomyelinase. Recombinant proteins based on the predicted M-SMase 5 and M-SMase 2 were incubated with colorimetric sphingomyelinase assay kit for 60 mins at 37ºC. Color changes in the reaction indicate sphingomyelinase activities. Hydrogen peroxide and sphingomyelinase from *Bacillis cereus* were included as positive controls. SpCas9 protein and buffer are used as negative control.

## Supplementary Tables

**Supplementary Table 1.** Nucleotide sequences of metagenomic SMases (M-SMases)

| SMase | nucelotide sequence |
| --- | --- |
| M-SMase1 | ATGAACCTAAAAACTGCCTTAAGCTTGGCGCTCAGTGCTAGCTTAGCATCCTCTTTAGTTATGGCTATGTCCGACAGCCCAACGGCTGTGGAAGCGCCACTAACACCCATACAGCCTCCCGTAAGTGAAGAAAGCTCAGGCCTAGCCTACGCTGATGATTTTCGCTTATTAGCCTATAACGTTTATATGTTGCCAGAGGTCATCGCCAACTGGAACCACGCTGGTCGCGCACAAATGATTGCCGACTCAGACTTGGTAAAAGGACATGACGCCATCTTATTGCAAGAACTGTTCGATAACGGCCCAGCCGATATCCTTTTAAACGGCTTAAAAGCAGAATACCCATACCAAACACAAGTACTTGGCCGCACTAAAAGTGGCTGGGATGCCACACTTGGTTCATACGCTGAGGGCACAATAGAAGACGGTGGTGTAGCCATTGTCAGCCGCTGGCCGATCGAAGAACAAATTCAATACGTATACAAGCAAGGCTGTGGTGCCGATTATTTAGCCAACAAAGGTTTTGTTTATGCGCGTATTGATAAAAACGGTGCCGATTACCACGTTATCTCAACCCACGTACAAGCCGAAGACAGTGCCTGTGATAATCCAGCCGCAACGCGTAAAAGCCAATTCATAGAAATGCAAAACTTCATTGCTAGCCACAATATTCCGGCAGACGAAGTAGTGTTTATGGGCGGCGACTTTAACGTGATTAAAGGCACCGATGAATACCCAGACATGCTAGAAACACTGAAAGCCAGCGCACCCGATGCGTATGCCGGTTTCGATACCAGCTGGGACCCAGAATCAAACGGCATTGCTGCGTATAACTACCCAGATCTGCCGAGTGAATACCTAGATTACATCTTTGTGTCGCGCGATCATGCACAGCCAACTCATTGGCATAACCAATCGTTAGACGTTACCTCACCAGGTAGCTGGGCAGTGGGTAACTACAAATACCAAGAACTGTCCGATCACTACCCGATTGCGGGTTTCTCGTACGCTAACGCAAGCACCAAAACCGAAAGTTATCGTGCGGTGAATAACCCATACCAAGGCGTTCAATTTCAGAACAAAGCCAACGGTAAATATATTCGCATAGACCCTAATAATGCCGACGGTTGGATCACAGTAAAAGGCGAAGCCAGCGACCCAGCAACGGTCATGAACATGGACAACTGGTATCCAAAGAATCGTGCATTTTGTATTCGCAACGACGACTGGATTCAAGTACAAGGCGCACAGCGTGAAGGCTCTTACTGGAACTGGTACTTAGGTGGCGGTGGTGGTAACTACGCGTACTTTACTAAACCAGACAACGCCTCTAATAAACTGCGTGTGCGCATTCTTAATGACGATGGCGACTGCCTGAAAGACGGCGACCAAATTGCTTTTGTTGATCGCAGCACCGTTAACGGCGTCGATTATTATCTACAGCGCTGGCCTTCAGGCTCTTGGCAGGATTACCTGTACCTGTGGTCAAACACAGTAGGGGATAACGAAACCTTCACGATTCATATGGGGCAGGCAGAAAAACAAAACTTTGCGCCAAGCCTACGTTATGCGCAATAG |
| M-SMase2 | ATGAAATTAAAAACTGCTTTGAGCGTCACGCTCAGTGCCTGTTTATCAGCGCCACTAGCCATGGCCATGTCAAACAGCCCAACCGTTGCCGACAGCCCCGTGGTGCCACAACCCACACAACCAGAAATGAGCCAAGGCTTAGCCTACGCAGACGATTTCCGCTTGTTGGCCTACAACGTTTATATGCTGCCAGAGCTGATCGCCAACTGGAATCACCAAGGCCGTGCAGAACTCATCGCAAACGCCGATTTCATGAAAGGCCATGATGCAATTTTACTGCAAGAACTGTTCGACAACGCACCCGCCAGCACAGTATTAAACGGCTTAAAAGCAGAATACCCTTATCAAACACAAGTACTAGGCCGAACTAAAGACGGCTGGGATGCCACCTTGGGCGCATACAGTGCCAGTTCCGTAGAAGACGGTGGCGTGGCTATTGTTAGCCGTTGGCCTATCGAAGAACAAATTCAATACGTTTATGCCCAAGGCTGTGGCGCAGATTTTTTTGCTAATAAAGGCTTTGTATATGCACGGGTTAATAAAAACGGCGCAGACTACCACTTAATTTCTACCCACGCCCAAGCCGAAGACAGCGCCTGCGACAACCCAGCGGCAACGCGTAAAAGCCAATTTGCAGAAATGCAAAGCTTCATCGCCAACCACAACATACCCGCAGACGAAGTGCTATTTTTAGGCGGTGATTTTAATGTTATTAAAGGCACCGACGAATACCCCGACATGCTTGAAACCTTAAAGGCCAGCGATCCCAATGCTTACGCCGGATTCAGCACCAGCTGGGACCCAAAATCTAACGGCATTGCAGCGTACAACTACCCAGACCTACCCAGTGAATACCTGGATTACATCTTTGTATCGCGCGACCACGCCCAACCAACTTATTGGCACAACCAATCGTTAGACGTAACATCATCGTTTAGCTGGTCGGTTGGCAACTATAAATACCAAGAACTGTCTGACCATTACCCGATTGCAGGATTCTCGTACGCTAATGAAAACACCCGCACCGAAAGCTACCGTGCGGTAAATAACCCTTACCAAAACGTACAGTTTCAAAACAAAGCCAATGGTAAATACATTCGTATCGACACCAATAACAACGACGGCTGGTTAACTGTAAAAGGTGACGTAAACGACCCAGCAACCGTAATGAACCTAGACAACTGGTACCCCAAAAACCGTGCTTTTTGTATTCGTAACGACGACTGGATTCAAGTGCAGGGCGCACAACGCGAAGGTTCTTACTGGAACTGGTGGTTAGGCGGCGGTGGCGGCAACTACGCCTTCTTCACTAAGCAAGACAACGCCTCTAACAAGCTGCGCGTAAGAATACTCAACGACGACGGCGACTGCCTAAAAAATGGCGACCAAATTGCCTTTGTTGATCGCAGCACGGTAAATGGAAGAGATTACTACCTACAGCGCTGGCCTTCTGGTACATGGCAAGATCACCTGTATTTATGGTCTAACTCAGTAGGGGATAACGAAACCTTTACCGTACGCATGGGGCAGGCAGAAAAGCAAAACTTTGTACCTAACTTACGCTATGCACAGTAA |
| M-SMase3 | ATGAATCTTAAAACCACCTTATGCCTCGCGGTCAGCGCGACGCTATCGTCGGCGATGCTCAGTACACCAGTACTGGCCATGTCAGACACTCCAACAGCTACTCCAGTCGTTGAACCGGCACCGACTACCCCAGTTGAAAACACAGGTCTGGCCTATGCTGACGACTTCCGTCTATTAGCCTACAACGTCTATATGTTGCCAGAGTTATTCGCCGGTGACTGGAGTCATGAGCAACGCGCTCAAATGATTCCTCAGTCAGAAGTCGTTCAAGGCCAAGACGCCATTCTATTGCAAGAGCTGTTTGATAATGGCCCTGCAACCACACTGCTAAATGGATTAAAAGCTGACTACCCATACCAAACTCCGGTAATGGGCCGCTCTAAAAGTGGTTGGGACGCAACCTTAGGTGCTTACGCCGACTTGTCCCCAGAGGATGGTGGTGTCGCCATTGTAAGCCGATGGCCAATCACAGAACAAATTCAATACGTTTATAAAGACGCCTGTGGGGCCGATTACCTGTCTAATAAAGGCTTTGTTTACGCTCGCATTGATAAAAATGGCGCGGCTTATCATTTAATCAGTACCCATGCTCAAGCAGAAGATAGTGCGTGTACGGATCCTGCGGCAACCCGTAAGAGCCAGTTCACCGAAATGCAAAACTTCATCGCCGGACATAATATTCCAGCGGACGAAGTGGTCTTCATGGGTGGTGATTTTAATGTAATTAAAGGCACAGCAGAATACCCAGACCTAATCGAAACATTACAAGTCAGTGAGCCAGACGCCTATGCCGGTTTCTCAACCAGCTGGGACCCTGAATCGAATGGAATGGCGGCGTATAATTACCCTGATCTGCCAAGTGAATACCTAGATTACATCTTTGTATCGCGTAATCACGCACAGCCTAGCCACTGGCACAACCAATCGTTGGATGTAACCTCAACTAATTGGAGTGCAGGTAACTACCGCTTCCAAGAGTTGTCTGATCACTACCCAATTGCCGGTTTCTCCTACGCGAACGAAAATACCCGCACTGAAAGCTTCCGCGCGGTGAACAATCCTTACCAAGGGGTTCAATTTCAGAACAAAGCCAATGGCAAATATATCCGTATTGATGCCAATGACAGTAATGGGTGGTTAACCGTGAAAGGTGAAGCCAATGACCCAGCGACTGTGATGAACATGGATAACTGGTACCCGAAAAATCGTGCTTTCTGTATCCGCCACGACGATTGGATTCAAGTGCAAGGTGCACAGCGTGCAGGTTCTTATTGGAATTGGTGGTTAGGAGGCGGTGGTGGTAACTACGCTTACTTCACCAAGCCTGATAACGCATCAAATAAACTGCGTATTCGTATCTTAAATGATGACGGCGATTGCTTAAAAGATGGTGACCAGATTGCCTTTGTCGATCGTAGCACGGTAAACGGTGTCGATTACTTCTTACAGCGCTGGCCTTCAGGTTCTTGGCAGGATTACCTGTACCTATGGTCTAATTCGGTAGGTGATAATGAAACCTTTACTGTTCATATGGGTCAAGGAGAGAAGCAGGACTTTACTCCGAACCTGAGATACGCACAGTAA |
| M-SMase4 | ATGAACTTAAAAACTGCCTTATGCCTCGCAGTCAGTGCGACGCTTACGTCGACCATGGCTTTCGCCATGTCAGACAGCCCCTCAACTGAGTCCTCAACCAATGCCTCCGCAGAGCCGGCTAATGGCTCCTCTAATATCAGCAGTGGTTTAGCCTATGCCGACGATTTTCGTCTATTAGCCTATAACGTCTACATGTTGCCAGAAGCCTTAGCCAACTGGAATCATGAAGGTCGTGCTCAATTAATAGCCCAATCTGAGGTTGTTCAAGGCCACGATGCTATTATGCTGCAAGAAATATTTGATAATGGCCCAGCAGCGACCTTATTAAATGGCTTAAAAGCTGAGTATCCGCACCAAACTCCCGTGCTTGGGCGCACTAAAGATGGCTGGGATGCGACGCTTGGGGCGTACGCCAATGCTTCACCAGAAGATGGAGGTGTTGCCATTGTTAGCCGCTGGCCCATTGAAGAGCAAATTCAATACGTTTATAAAAACGGCTGTGGTGCTGATTACCTCGCGAATAAAGGGTTTGTTTATGCCCGCATTGATAAAAATGGCGCAAACTACCATGTGATTGGTACCCATGCTCAAGCCGAAGATGGTGCTTGTGACGATCCGGCTGCAACGCGTAAAACACAATTTATCGAGATGCAAAACTTTATCGCTCAGCATAATATTCCAGCAGACGAAGTCGTCTTTATGGGAGGGGATTTTAATGTCATTAAAGGCAGCACCGAATATCCTGACATGCTAGAAACACTGCAGGTCAGTGACATTGATACCTATGGGGGTTTTGAAACCAGCTGGGACCCAGAGTCTAATGGTATTGCCGCTTATAATTACCCAGATTTACCGAGTGAATACCTAGATTACATCTTTGTCTCGCGCAACCATGCCCAGCCTAGTCATTGGCATAACCAGTCGTTAGATGTGACTTCGACTAACTGGTCTGTGGGGAATTATCGCTTCCAAGAATTATCCGATCATTACCCGATTGCGGGCTTCTCGTATGCGAATGCCGAAACGCGCACTGAAAGCTTCCGTGCGGTGAATAACCCATACCAGGGTATCCAGTTGCAAAACAAAGCCAATGGTAAGTTTATTCGTATCGACCCGAATAACGATAATGGCTGGATCACAGTAAAAGGTGAAGCCAACGATCCTGCGACAGTTATGAATATGGATAACTGGTACCCTAAAAACCGTGCTTTCTGTATTCGCAACGACGATTGGATTCAAGTACAAGGCGCTATGCGTGAAGGCTCTTATTGGAACTGGTGGTTAGGGGGCGGTGGTGGAAATTATGCCTTCTATACTAAGCCCGATGATGCCTCTAATAAGCTACGCATTCGCATTTTGAACGATGATGGTGATTGTTTACAAAACGGTGATCAGGTGGCTTTTGTTGATCGCAGTACCGCAAATGGCGTGGATTATTATCTACAGCGCTGGCCATCAGGTTCTTGGCAAGATTATCTATACCTGTGGTCTAACTCTGTAGGTGATAACGAAACCTTTACGGTGCATATGGGGCAAGCGCAAAAGCAAGATTTCACACCGAATCTACGTTACGCTCAATAA |
| M-SMase5 | ATGGTGAAAAAAACAAAATCCAATTCACTAAAAAAAGTTGCAACACTTGCATTAGCAAATTTATTATTAGTTGGTGCACTTACTGACAATAGTGCCAAAGCCGAATCTAAGAAAGATGATACTGATTTGAAGTTAGTTAGTCATAACGTTTATATGTTATCGACCGTTTTGTATCCGAATTGGGGGCAATATAAACGTGCTGATTTAATCGGACAATCTTCTTATATTAAAAATAATGATGTCGTAATATTCAATGAAGCATTTGATAATGGTGCATCAGATAAATTATTAAGTAATGTAAAAAAAGAATATCCTTATCAAACACCTGTACTAGGTCGTTCTCAATCAGGGTGGGACAAAACTGAAGGTAGCTACTCATCAACTGTTGCTGAAGATGGTGGCGTAGCGATTGTAAGTAAATATCCTATTAAAGAAAAAATCCAGCATGTTTTCAAAAGCGGTTGTGGATTCGATAATGATAGCAACAAAGGCTTTGTTTATACAAAAATAGAGAAAAATGGTAAGAACGTTCACGTTATCGGTACACATACACAATCTGAAGATTCACGTTGTGGTGCTGGACATGATCGAAAAATTAGAGCTGAACAAATGAAAGAAATCAGTGACTTTGTTAAAAAGAAAAATATCCCTAAAGATGAAACGGTATATATAGGTGGCGACCTTAATGTTAATAAAGGTACTCCAGAGTTCAAAGATATGCTTAAAAACTTGAATGTAAATGATGTTCTATATGCAGGTCATAATAGCACATGGGACCCTCAATCAAATTCAATTGCGAAATATAATTACCCTAATGGTAAACCAGAACATTTAGACTATATATTTACAGATAAAGATCATAAACAACCAAAACAATTAGTCAATGAAGTTGTGACTGAAAAACCTAAGCCATGGGATGTATATGCGTTCCCATATTACTACGTTTACAATGATTTTTCAGATCATTACCCAATCAAAGCCTATAGTAAATAG |
| M-SMase6 | ATGAAACGAGGTGTAACAATATTGAATTGGCAACGTAAATGTATACTAACTACTTTGTTGGTTTTAAGTAGTTTATTTTTAGTATTTTCGACTATCACATATGCGAGTGAACGTGATTTTAAAGACAGTCTTAAAATCACTACACACAACGTGTATTTCTTACCTACTGCTATCTACCCTAATTGGGGACAATCTCAGCGCGCTGATTTAATTTCAAAAGCAGATTACATTCAAAATCAAGATGTCGTGATTCTAAATGAATTATTTGATAAAAAAGCTTCAAAAAGATTGTTAACACGTCTACATTCACAGTACCCCTATCAAACACCTATCGTTGGTAAGGGTACAGAAGGTTGGCAAAATACTTCTGGTACTTATAGAAAAATTAAAAAAGTAAGTGGTGGCGTTGGTATTGTGAGTAAATGGCCTATCGTACAACAAGAACAACATATTTATAAAAAAGGCTGTGGGGCTGATATGGCAGGTAATAAAGGCTTTGCCTACATTAAAATTAATAAGAATGGCAAATACCACCACATTATCGGAACACATCTACAAGCTGAAGATCCAACATGTTTTAAAGGAAAAGATAAAGATATTAGACAGAGTCAAATGAGTGAAATTAAACAGTTTATCAAAGACAAGAATATCCCTAAAAATGAACCCGTCTATATCGGTGGTGACTTAAATGTCATTAAAGATTCAGATGAATATCAACAAATGTCAAATAACTTAAATGTTTCATTATCTACTCAATTCGATGGTAATGCATATAGTTGGGATACTAGCAGTAATAGTATTGCGAAATATAATTATCCTAAATTAGAACCTCAACACTTAGATTATATTTTATTAGATCGTGACCATGCACAACCAAGCTCATGGCATAATGATACACATAGAGTGAAGTCACCAGAATGGTCTGTGAAATCTTGGGGAAAAACATACAAATACAATGATTACTCAGATCATTACCCACTCTCAGGCTATGCATCAAATGAATAG |

**Supplementary Table 2.** Amino acid sequences of metagenomic SMases (M-SMases)

| SMase | protein sequence |
| --- | --- |
| M-SMase1 | MNLKTALSLALSASLASSLVMAMSDSPTAVEAPLTPIQPPVSEESSGLAYADDFRLLAYNVYMLPEVIANWNHAGRAQMIADSDLVKGHDAILLQELFDNGPADILLNGLKAEYPYQTQVLGRTKSGWDATLGSYAEGTIEDGGVAIVSRWPIEEQIQYVYKQGCGADYLANKGFVYARIDKNGADYHVISTHVQAEDSACDNPAATRKSQFIEMQNFIASHNIPADEVVFMGGDFNVIKGTDEYPDMLETLKASAPDAYAGFDTSWDPESNGIAAYNYPDLPSEYLDYIFVSRDHAQPTHWHNQSLDVTSPGSWAVGNYKYQELSDHYPIAGFSYANASTKTESYRAVNNPYQGVQFQNKANGKYIRIDPNNADGWITVKGEASDPATVMNMDNWYPKNRAFCIRNDDWIQVQGAQREGSYWNWYLGGGGGNYAYFTKPDNASNKLRVRILNDDGDCLKDGDQIAFVDRSTVNGVDYYLQRWPSGSWQDYLYLWSNTVGDNETFTIHMGQAEKQNFAPSLRYAQ |
| M-SMase2 | MKLKTALSVTLSACLSAPLAMAMSNSPTVADSPVVPQPTQPEMSQGLAYADDFRLLAYNVYMLPELIANWNHQGRAELIANADFMKGHDAILLQELFDNAPASTVLNGLKAEYPYQTQVLGRTKDGWDATLGAYSASSVEDGGVAIVSRWPIEEQIQYVYAQGCGADFFANKGFVYARVNKNGADYHLISTHAQAEDSACDNPAATRKSQFAEMQSFIANHNIPADEVLFLGGDFNVIKGTDEYPDMLETLKASDPNAYAGFSTSWDPKSNGIAAYNYPDLPSEYLDYIFVSRDHAQPTYWHNQSLDVTSSFSWSVGNYKYQELSDHYPIAGFSYANENTRTESYRAVNNPYQNVQFQNKANGKYIRIDTNNNDGWLTVKGDVNDPATVMNLDNWYPKNRAFCIRNDDWIQVQGAQREGSYWNWWLGGGGGNYAFFTKQDNASNKLRVRILNDDGDCLKNGDQIAFVDRSTVNGRDYYLQRWPSGTWQDHLYLWSNSVGDNETFTVRMGQAEKQNFVPNLRYAQ |
| M-SMase3 | MNLKTTLCLAVSATLSSAMLSTPVLAMSDTPTATPVVEPAPTTPVENTGLAYADDFRLLAYNVYMLPELFAGDWSHEQRAQMIPQSEVVQGQDAILLQELFDNGPATTLLNGLKADYPYQTPVMGRSKSGWDATLGAYADLSPEDGGVAIVSRWPITEQIQYVYKDACGADYLSNKGFVYARIDKNGAAYHLISTHAQAEDSACTDPAATRKSQFTEMQNFIAGHNIPADEVVFMGGDFNVIKGTAEYPDLIETLQVSEPDAYAGFSTSWDPESNGMAAYNYPDLPSEYLDYIFVSRNHAQPSHWHNQSLDVTSTNWSAGNYRFQELSDHYPIAGFSYANENTRTESFRAVNNPYQGVQFQNKANGKYIRIDANDSNGWLTVKGEANDPATVMNMDNWYPKNRAFCIRHDDWIQVQGAQRAGSYWNWWLGGGGGNYAYFTKPDNASNKLRIRILNDDGDCLKDGDQIAFVDRSTVNGVDYFLQRWPSGSWQDYLYLWSNSVGDNETFTVHMGQGEKQDFTPNLRYAQ |
| M-SMase4 | MNLKTALCLAVSATLTSTMAFAMSDSPSTESSTNASAEPANGSSNISSGLAYADDFRLLAYNVYMLPEALANWNHEGRAQLIAQSEVVQGHDAIMLQEIFDNGPAATLLNGLKAEYPHQTPVLGRTKDGWDATLGAYANASPEDGGVAIVSRWPIEEQIQYVYKNGCGADYLANKGFVYARIDKNGANYHVIGTHAQAEDGACDDPAATRKTQFIEMQNFIAQHNIPADEVVFMGGDFNVIKGSTEYPDMLETLQVSDIDTYGGFETSWDPESNGIAAYNYPDLPSEYLDYIFVSRNHAQPSHWHNQSLDVTSTNWSVGNYRFQELSDHYPIAGFSYANAETRTESFRAVNNPYQGIQLQNKANGKFIRIDPNNDNGWITVKGEANDPATVMNMDNWYPKNRAFCIRNDDWIQVQGAMREGSYWNWWLGGGGGNYAFYTKPDDASNKLRIRILNDDGDCLQNGDQVAFVDRSTANGVDYYLQRWPSGSWQDYLYLWSNSVGDNETFTVHMGQAQKQDFTPNLRYAQ |
| M-SMase5 | MVKKTKSNSLKKVATLALANLLLVGALTDNSAKAESKKDDTDLKLVSHNVYMLSTVLYPNWGQYKRADLIGQSSYIKNNDVVIFNEAFDNGASDKLLSNVKKEYPYQTPVLGRSQSGWDKTEGSYSSTVAEDGGVAIVSKYPIKEKIQHVFKSGCGFDNDSNKGFVYTKIEKNGKNVHVIGTHTQSEDSRCGAGHDRKIRAEQMKEISDFVKKKNIPKDETVYIGGDLNVNKGTPEFKDMLKNLNVNDVLYAGHNSTWDPQSNSIAKYNYPNGKPEHLDYIFTDKDHKQPKQLVNEVVTEKPKPWDVYAFPYYYVYNDFSDHYPIKAYSK |
| M-SMase6 | MKRGVTILNWQRKCILTTLLVLSSLFLVFSTITYASERDFKDSHKITTHNVYFLPTAIYPNWGQSQRADLISKADYIQNQDVVILNELFDKKASKRLLTRLHSQYPYQTPIVGKGTEGWQNTSGTYRKIKKVSGGVGIVSKWPIVQQEQHIYKKGCGADMAGNKGFAYIKINKNGKYHHIIGTHLQAEDPTCFKGKDKDIRQSQMSEIKQFIKDKNIPKNEPVYIGGDLNVIKDSDEYQQMANNLNVSLPTQFDGNAYSWDTSSNSIAKYNYPKLEPQHLDYILLDRDHAQPSSWHNDTHRVKSPEWSVKSWGKTYKYNDYSDHYPLSGYASNE |

**Supplementary Table 3.** Putative SMases identified from microbiome data

| SMase | sequence name | length (aa) | homologous protein | source organism of homologous protein | seq. id. (%) |
| --- | --- | --- | --- | --- | --- |
| M-SMase1 | ERR2094173_417459_5063_6640_+ | 525 | sphingomyelin phosphodiesterase (CCK76853.1) | *Oleispira antarctica* RB-8 | 78.05 |
| M-SMase2 | WD2B1711_3438739_1427_3001_+ | 524 | sphingomyelin phosphodiesterase (CCK76853.1) | *Oleispira antarctica* RB-8 | 75.52 |
| M-SMase3 | WD2B1711_4256387_1411_2994_+ | 527 | sphingomyelin phosphodiesterase (CCK76853.1) | *Oleispira antarctica* RB-8 | 85.36 |
| M-SMase4 | WD4B1711_269829_3641_5221_- | 526 | sphingomyelin phosphodiesterase (CCK76853.1) | *Oleispira antarctica* RB-8 | 78.24 |
| M-SMase5 | SRS011105_contig-100_14.14_28828_29823_+ | 330 | sphingomyelin phosphodiesterase (WP_001652363.1) | *Staphylococcus aureus* | 100 |
| M-SMase6 | SRS018978_contig-100_70.14574_9877_10881_+ | 334 | sphingomyelin phosphodiesterase (MBM0781907.1) | *Staphylococcus epidermidis* | 100 |
| - | SRS016752_contig-100_1754.1754_58_1062_+ | 334 | sphingomyelin phosphodiesterase (KAA9275130.1) | *Staphylococcus epidermidis* | 99.7 |
| - | SRS022006_contig-100_2984.2984_56_1060_+ | 334 | sphingomyelin phosphodiesterase (KAA9275130.1) | *Staphylococcus epidermidis* | 99.4 |
| - | SRS023847_26_488_1492_- | 334 | sphingomyelin phosphodiesterase (EES57778.1) | *Staphylococcus epidermidis* | 99.7 |
| - | SRS024424_contig-100_2011.2011_121_1125_+ | 334 | sphingomyelin phosphodiesterase (EES57778.1) | *Staphylococcus epidermidis* | 100 |
| - | SRS044474_contig-100_1760.1760_316_1320_- | 334 | sphingomyelin phosphodiesterase (AXE40331.1) | *Staphylococcus epidermidis* | 100 |
| - | SRS063035_contig-100_2.2_9038_10042_+ | 334 | sphingomyelin phosphodiesterase (KAA9252073.1) | *Staphylococcus epidermidis* | 100 |
| - | SRS020261_153_558_1562_+ | 334 | sphingomyelin phosphodiesterase (NAM29562.1) | *Staphylococcus epidermidis* | 99.4 |
| - | SRS024482_731_6729_7733_+ | 334 | sphingomyelin phosphodiesterase (MBM0873408.1) | *Staphylococcus epidermidis* | 99.7 |
| - | SRS020263_1595_176_1180_+ | 334 | sphingomyelin phosphodiesterase (KAB2191979.1) | *Staphylococcus epidermidis* | 98.2 |

**Supplementary Table 4.** Sequence conservation of metal-binding sites in known and putative SMases

| group | SMase | central metal-binding | | | | | | edge metal-binding | | | |
| --- | --- | --- | --- | --- | --- | --- | --- | --- | --- | --- | --- |
| Known | Sa-SMase | N16 | E53 | D194 | N196 | D284 | H285 | F55 | N57 | E98 | D99 |
|  | Bc-SMase | N16 | E53 | D195 | N197 | D295 | H296 | F55 | N57 | E99 | D100 |
|  | Li-SMase | N51 | E88 | D229 | N231 | D324 | H325 | F90 | T92 | E133 | D134 |
|  | Sg-SMase | N57 | E94 | D235 | N237 | G330 | H331 | F96 | N98 | E139 | D140 |
| Metagenome | M-SMase1 | N59 | E95 | D234 | N236 | D326 | H327 | F97 | N99 | E140 | D141 |
|  | M-SMase2 | N58 | E94 | D233 | N235 | D325 | H326 | F96 | N98 | E139 | D140 |
|  | M-SMase3 | N61 | E98 | D237 | N239 | D328 | H329 | F100 | N102 | E143 | D144 |
|  | M-SMase4 | N61 | E97 | D236 | N238 | D327 | H328 | F99 | N101 | E142 | D143 |
|  | M-SMase5 | N49 | E86 | D227 | N229 | D321 | H322 | F88 | N90 | E131 | D132 |
|  | M-SMase6 | N49 | E86 | D227 | N229 | D322 | H323 | F88 | K90 | V131 | S132 |
| Bacterial complete genomes | *Staphylococcus* (n=80) | N:100% | E:100% | D:100% | N:100% | D:99%, E:1% | H:100% | F:100% | N:61%, K:19%, P:8%, H:5% , R:4% , E:3% , S:1% | E:69%, V:24%, S:4%, T:4% | D:61%, S:24%, N:15% |
|  | *Bacillus* (n=87) | N:100% | E:100% | D:100% | N:100% | D:100% | H:99%, Y:1% | F:100% | N:100% | E:100% | D:100% |
|  | *Listeria* (n=4) | N:100% | E:100% | D:100% | N:100% | D:100% | H:100% | F:100% | T:100% | E:100% | D:100% |
|  | *Streptomyces* (n=90) | N:100% | E:100% | D:100% | N:100% | D:100% | H:100% | F:100% | N:99%, D:1% | E:100% | D:100% |
|  | *Allokutzneria* (n=1) | N:100% | E:100% | D:100% | N:100% | D:100% | H:100% | F:100% | N:100% | E:100% | D:100% |
|  | *Arthrobacter* (n=1) | N:100% | E:100% | D:100% | N:100% | D:100% | H:100% | F:100% | N:100% | E:100% | D:100% |
|  | *Kitasatospora* (n=3) | N:100% | E:100% | D:100% | N:100% | D:100% | H:100% | F:100% | N:100% | E:100% | D:100% |
|  | *Lentzea* (n=1) | N:100% | E:100% | D:100% | N:100% | D:100% | H:100% | F:100% | N:100% | E:100% | D:100% |
|  | *Luteipulveratus* (n=1) | N:100% | E:100% | D:100% | N:100% | D:100% | H:100% | F:100% | N:100% | E:100% | D:100% |
|  | *Nocardia* (n=3) | N:100% | E:100% | D:100% | N:100% | D:100% | H:100% | F:100% | N:100% | E:100% | D:100% |
|  | *Nocardiopsis* (n=1) | N:100% | E:100% | D:100% | N:100% | D:100% | H:100% | F:100% | N:100% | T:100% | N:100% |
|  | *Nostoc* (n=1) | N:100% | E:100% | D:100% | N:100% | D:100% | H:100% | F:100% | N:100% | E:100% | D:100% |
|  | *Saccharothrix* (n=1) | N:100% | E:100% | D:100% | N:100% | D:100% | H:100% | F:100% | N:100% | E:100% | D:100% |
|  | *Salinispora* (n=2) | N:100% | E:100% | D:100% | N:100% | D:100% | H:100% | F:100% | N:100% | E:100% | D:100% |

**Supplementary Table 5.** Sequence conservation of the edge metal-binding site in SMases of *Staphylococcus* species

| species | F55 in Sa-SMase | N57 in Sa-SMase | E98 in Sa-SMase | D99 in Sa-SMase |
| --- | --- | --- | --- | --- |
| *S. agnetis* (n=2) | F:100% | E:100% | T:100% | N:100% |
| *S. argenteus* (n=2) | F:100% | N:100% | E:100% | D:100% |
| *S. aureus* (n=40) | F:100% | N:100% | E:100% | D:100% |
| *S. capitis* (n=2) | F:100% | K:100% | V:100% | S:100% |
| *S. caprae* (n=1) | F:100% | K:100% | V:100% | S:100% |
| *S. chromogenes* (n=3) | F:100% | R:100% | S:100% | N:100% |
| *S. delphini* (n=1) | F:100% | P:100% | E:100% | N:100% |
| *S. epidermidis* (n=11) | F:100% | K:100% | V:100% | S:100% |
| *S. felis* (n=1) | F:100% | S:100% | T:100% | N:100% |
| *S. lugdunensis* (n=4) | F:100% | H:100% | V:100% | S:100% |
| *S. lutrae* (n=1) | F:100% | N:100% | E:100% | D:100% |
| *S. pseudointermedius* (n=5) | F:100% | P:100% | E:100% | N:100% |
| *S. schleiferi* (n=4) | F:100% | N:100% | E:100% | D:100% |
| *S. schweitzeri* (n=1) | F:100% | N:100% | E:100% | D:100% |
